# An *in vitro* collagen gel contraction assay to assess the relaxing effect of potential pharmacological alternatives to oxytetracycline on foals’ tendons

**DOI:** 10.1038/s41598-026-49449-4

**Published:** 2026-04-25

**Authors:** Emmanuel Mathieu Cardinaux, Hilke Oltmanns, Karl Rohn, Jessica Meißner, Florian Geburek

**Affiliations:** 1 Clinic for Horses, University of Veterinary Medicine Hannover, Foundation, Hannover, Germany; 2 Department of Pharmacology, Toxicology and Pharmacy, University of Veterinary Medicine Hannover, Foundation, Hannover, Germany; 3 Institute of Biometry, Epidemiology, and Information Processing, University of Veterinary Medicine Hannover, Foundation, Hannover, Germany

**Keywords:** Oxytetracycline, Matrix-metalloproteinase inhibitors, Collagen gel contraction assay, Flexural limb deformities, Foals, Biological techniques, Diseases, Drug discovery, Medical research

## Abstract

Oxytetracycline has been successfully used to treat flexural limb deformities in foals. However, its antimicrobial properties and potential adverse side effects warrant the investigation of alternatives. Inhibition of matrix metalloproteinases (MMPs) or collagen cross-linking have been suggested as possible mechanisms of action. This study assessed the influence of potential alternative substances lacking antimicrobial properties on collagen gel contraction by juvenile equine myofibroblasts *in vitro*. Myofibroblasts were isolated from forelimb SDFTs and inferior check ligaments from foals euthanised for reasons unrelated to this study, and seeded in collagen gels containing oxytetracycline, MMP-inhibitors incyclinide, ilomastat, aprotinin, and pentoxifylline, β-aminopropionitrile fumarate (BAPN), and control medium. The gels were measured in photographs up to 96 h after release, and differences in contraction were evaluated. Compared to control, BAPN and incyclinide induced significant inhibition (*p* < 0.05) of collagen gel contraction by myofibroblasts at all time points, and the inhibition was more potent than that observed with oxytetracycline. Consequently, the inhibitory capacity of BAPN and incyclinide on myofibroblast-mediated collagen gel contraction, which demonstrates their effect on tractional structuring of collagen, makes them promising candidates as potential substitutes for oxytetracycline. Further investigations into their side effects, pharmacodynamics, pharmacokinetics, and application methods in juvenile equine patients are needed.

**Supplementary Information:**

The online version contains supplementary material available at 10.1038/s41598-026-49449-4.

## Introduction

Tetracyclines are broad-spectrum antibiotics that have demonstrated relevant properties unrelated to their antimicrobial effects^1^. They have been studied in human dentistry and oncology for their inhibitory effects on connective tissue breakdown by inhibiting matrix metalloproteinases (MMPs)^1,2^. Oxytetracycline (OTC), a first-generation tetracycline, has also been used with objective success as a therapeutic option for flexural limb deformities in foals^3^. The first observations of the relaxing effect of OTC on the muscle-tendon unit of foals affected by flexural limb deformities date back to the 1970s, and this effect was subsequently objectively described and confirmed radiographically and kinematically *in vivo*^3–5^.

However, the mechanism of action of OTC on the muscle-tendon unit is still incompletely understood. It has been hypothesized that the MMP-inhibitor properties of tetracyclines are involved, and Arnoczky et al. (2004) demonstrated a reduction in matrix metalloproteinase (MMP)-1 mRNA expression by myofibroblasts of foal tendons after exposure to OTC^6^. In contrast to this mechanism, research on the effect of OTC on the viscoelastic properties of rat tail tendon fascicles suggested similarities with the described effect of latyrogens on collagen cross-linking^7^. Inhibition of calcium-mediated muscle contraction through calcium chelation or neuromuscular blockage are other potential mechanisms mentioned in the literature^8,9^.

Following guidelines for the prudent use of antimicrobials^10^ and considering the potential adverse side effects of OTC, such as acute renal failure^11,12^, investigating alternatives without antimicrobial properties is desirable. Substances sharing the MMP-inhibitor properties of OTC, including the chemically modified tetracycline (CMT) incyclinide, ilomastat, pentoxifylline, and aprotinin, or showing inhibition of collagen cross-linking, such as β-aminopropionitrile fumarate (BAPN), have been tested *in vitro*, and their biocompatibility with juvenile equine myofibroblasts has been demonstrated^13^.

In addition to being biocompatible with the myofibroblasts of foals’ tendons and ligaments, a prerequisite for an alternative substance to OTC as therapy for flexural limb deformities is its relaxing effect on the muscle-tendon unit of foals. Collagen contraction assays have been used *in vitro* to simulate tissue contraction^14^. Skin fibroblasts incubated in collagen gels have been used in multiple studies to model wound contraction, and the influence of MMP-inhibitors on collagen gel contraction by periodontal ligament cells has been investigated^15,16^. Myofibroblasts, which are modified fibroblasts, comprise the primary cell population of juvenile tendons and ligaments within the flexor tendon unit, and collagen contraction assays have been successfully employed to simulate myofibroblast-mediated tractional structuring of collagen in juvenile tendons and ligaments^6,17^.

In this study, the influence of potential alternative substances to OTC, lacking antimicrobial properties and previously tested in viability and proliferation assays^13^, on collagen gel contraction by juvenile equine myofibroblasts was assessed *in vitro* to determine their potential relaxing effect on the juvenile equine muscle-tendon unit.

## Materials and methods

### Cell culture experiments

Tissue was harvested from foals euthanised in a clinical setting for reasons unrelated to this study. The foals were euthanised at the Clinic for Horses of the University of Veterinary Medicine Hannover and at the stud farm Gestüt Lewitz (Neustadt-Glewe, Germany) for clinical reasons, including pneumonia, neonatal maladjustment syndrome, gastrointestinal anomalies, *Rhodococcus equi* infection and peritonitis. Sedation was performed with xylazine (0.85 mg/kg bwt. i.v.; CP-Pharma Handelsgesellschaft mbH, Burgdorf, Germany) and butorphanol (0.02 mg/kg bwt. i.v; CP-Pharma Handelsgesellschaft mbH, Burgdorf, Germany), followed by induction into general anaesthesia using ketamine (2.5 mg/kg bwt. i.v.; Vetoquinol GmbH, Ismaning, Germany) and diazepam (0.05 mg/kg bwt. i.v.; TMV Tiergesundheit GmbH, Berlin, Germany) before euthanasia was performed using pentobarbital (100 mg/kg i.v.; CP-Pharma Handelsgesellschaft mbH, Burgdorf, Germany). All methods were performed in accordance with the relevant guidelines and regulations, and the experiments were approved as an *in vitro* study without the use of experimental animals by the University of Veterinary Medicine Hannover’s animal welfare officer.

Juvenile equine myofibroblasts were isolated and cultured as previously described^13^. Briefly, the superficial digital flexor tendon (SDFT) and the respective accessory ligament of the deep digital flexor tendon (ALDDFT) of the forelimbs of 6 warmblood foals, aged between 2 and 77 days (median 11 days), were aseptically collected within 6 h of death and stored in Dulbecco’s modified eagle medium (DMEM) (Carl Roth GmbH, Karlsruhe, Germany) with 1% penicillin/streptomycin (10,000 I.U./mL / 10,000 µg/mL, Bio&Sell GmbH, Feucht, Germany) for transport. After being washed in phosphate-buffered saline (PBS), the tendons and ligaments were cut into 0.25 cm^3^ pieces with a scalpel blade. The obtained pieces were then incubated with 3 mg/mL collagenase A (Roche Diagnostics GmbH, Mannheim, Germany) in DMEM containing 1% penicillin/streptomycin at 37 °C with 5% CO_2_ for 16–20 h. The cell suspension was then filtered through 70 μm and 40 μm nylon mesh filters and centrifuged at 1,000 × g for 5 min. The supernatant was removed, and the cell pellet was resuspended in DMEM with 1% penicillin/streptomycin and 10% fetal bovine serum (Bio&Sell GmbH, Feucht, Germany). The cells were cultured in 25 cm² tissue culture flasks at 37 °C with 5% CO₂ as a monolayer at a density of 1 million cells/flask.

### Drugs

The drugs used are presented in Table 1. Chosen concentrations reflect concentrations reported in the literature, either in *in vitro* experiments^1,18–21^ or in clinical applications^22–26^. These concentrations, except OTC at 125 µg/mL, were tested in a precedent biocompatibility study^13^.

**Table 1:** Drugs with corresponding solvents and concentrations used in the study.

Drug	Solvent	Concentration
Oxytetracycline^1^	Dulbecco’s modified eagle medium (DMEM)	75 and 125 µg/mL
Incyclinide^2^	Dimethyl sulfoxide (DMSO) solution in Dulbecco’s modified eagle medium (DMEM)	7.5 µg/mL in 0.375% DMSO
Ilomastat^3^	DMSO solution in DMEM	20 µg/mL in 1% DMSO
Aprotinin^4^	DMEM	200 µg/mL
Pentoxifylline^5^	DMEM	12 µg/mL
BAPN^6^	DMEM	1000 and 3000 µg/mL

^1^Sigma-Aldrich, Steinheim, Germany.

^2^MedChemExpress, Monmouth Junction USA.

^3^MedChemExpress, Monmouth Junction, USA.

^4^MedChemExpress, Monmouth Junction, USA.

^5^MedChemExpress, Monmouth Junction, USA.

^6^MedChemExpress, Monmouth Junction, USA.

### Collagen gel contraction assay

**Fig. 1:**
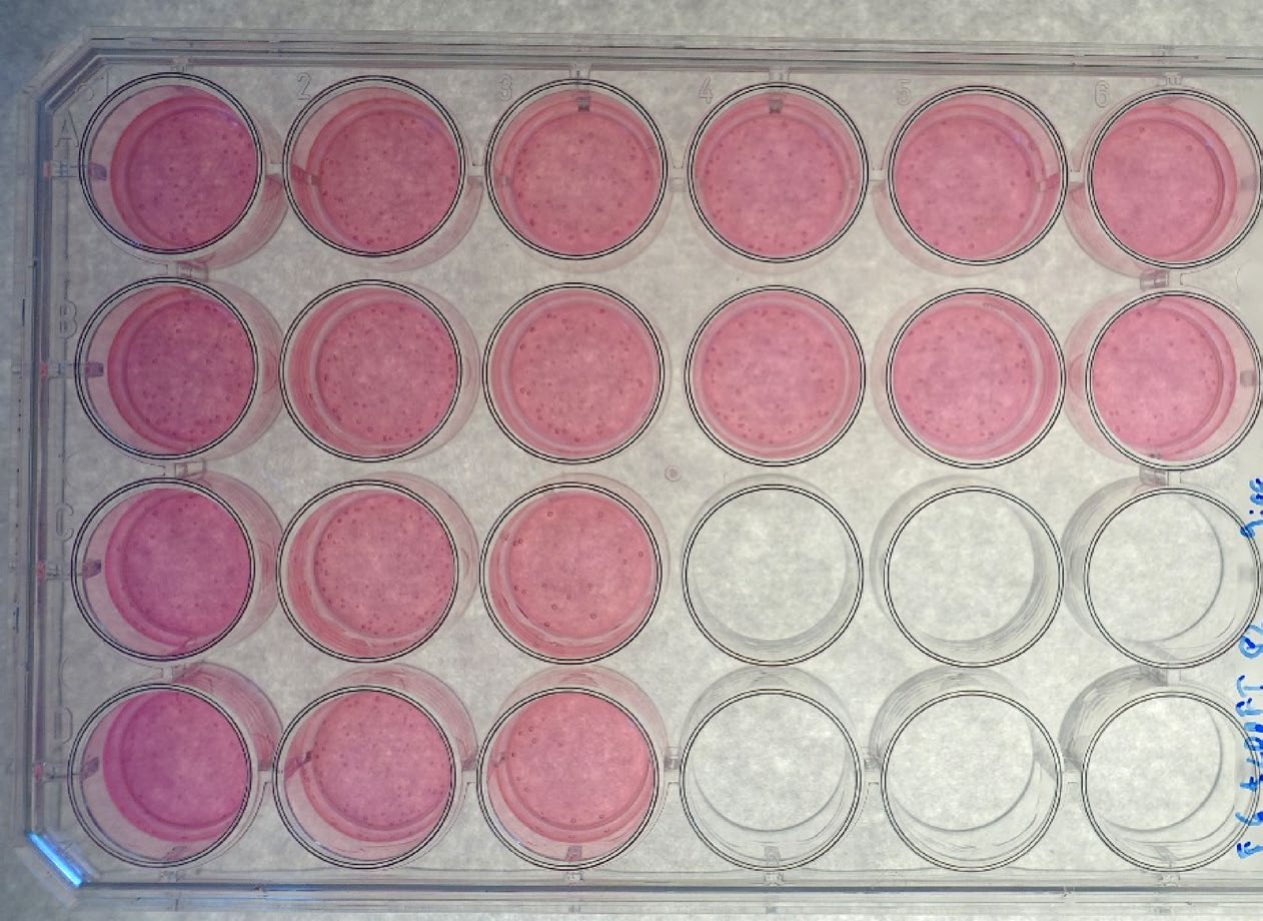
Representative photograph of a 24-well plate containing collagen gels seeded with myofibroblasts of an accessory ligament of the deep digital flexor tendon (ALDDFT) and incubated with Dulbecco’s modified eagle medium (A2 and B2), β-aminopropionitrile fumarate (BAPN) at 3000 µg /mL (C1 and D1), pentoxifylline at 12 µg/mL (C2 and D2), oxytetracycline (OTC) at 75 µg/mL (A3 and B3) and at 125 µg/mL (C3 and D3), incyclinide at 7.5 µg/mL (A4 and B4), ilomastat at 20 µg/mL (A5 and B5) and aprotinin at 200 µg/mL (A6 and B6) at the time of release; wells A1 and B1 contain collagen gels not incubated with any cells.

A collagen contraction assay was performed as previously described, with some modifications^6,27^. In brief, after preliminary tests to determine optimal composition, collagen gels (rat tail collagen type I, [2.4 mg/mL], Merck KGaA, Darmstadt, Germany and Corning Inc., New York, USA), containing either DMEM alone, OTC or one of the test substances (incyclinide, ilomastat, aprotinin, pentoxifylline or BAPN) at the concentrations listed in Table 1, were seeded with juvenile equine myofibroblasts (200,000 cells/mL, passage 2) and placed in a 24-well plate (500 µL/well). After 20 min, 500 µL of DMEM alone or DMEM containing the corresponding substance (Table 1) was added to each well. After incubation at 37 °C with 5% CO_2_ for 19 h, the gels were released from the bottom of the wells, by running the tip of a 200-µL pipet tip along the gel edges and gently lifting the edges of the gel with the pipet tip (Fig. 1). The collagen gel contraction assay was performed in six biological replicates and two technical replicates for each compound, and each tendon/ligament type (SDFT and ALDDFT) as well as one or two technical replicates for DMEM and OTC at a concentration of 125 µg/mL. Technical replicates were averaged, and the resulting mean value was used for statistical analysis. In a supplementary experiment, a collagen gel contraction assay was performed in three biological replicates for each tendon/ligament type (SDFT and ALDDFT) and one technical replicate using gels containing BAPN at a concentration of 1000 µg/mL and DMSO at 1%, as well as DMSO 0.375% in one biological replicate and one technical replicate for ALDDFT only.

### Gel Imaging and measurement

**Fig. 2:**
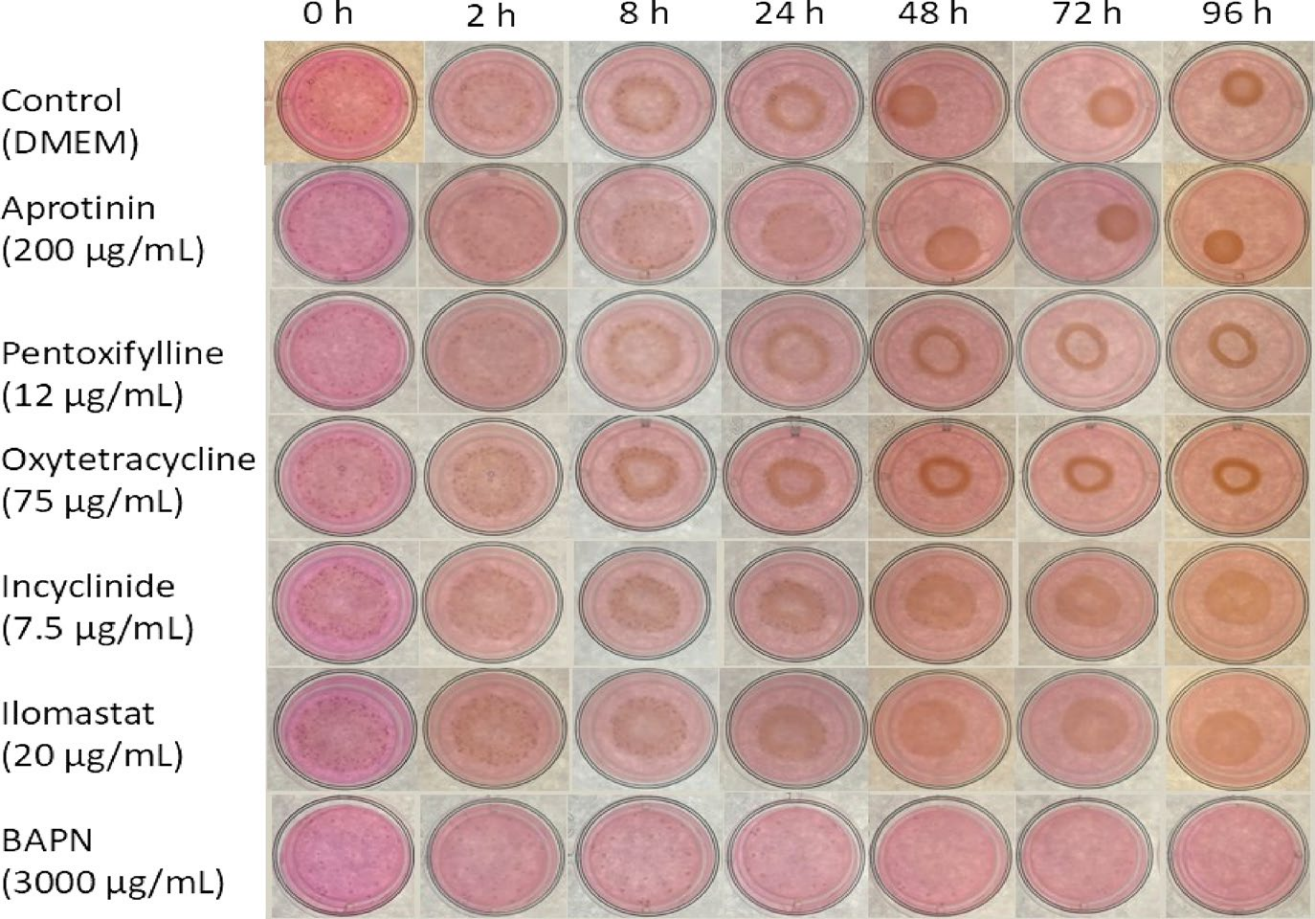
Representative serial photographs of collagen gels seeded with myofibroblasts of an accessory ligament of the deep digital flexor tendon (ALDDFT) and incubated with Dulbecco’s modified eagle medium (DMEM), aprotinin (200 µg/mL), pentoxifylline (12 µg/mL), oxytetracycline (OTC) (75 µg/mL), incyclinide (7.5 µg/mL), ilomastat (20 µg/mL) or β-aminopropionitrile fumarate (BAPN) (3000 µg/mL); time points (0, 2, 8, 24, 48, 72 and 96 h) are indicated above.

The gels were imaged after release using digital photography with a Sony RX100 III (Sony, Tokyo, Japan) with a constant digital zoom and a distance of 29 cm. The 24-well plate was positioned on a light box. Images were captured at release as well as 2, 4, 6, 8, 24, 48, 72 and 96 h after the release of the gels (Fig. 2). The gel surface area was measured using ImageJ (National Institute of Health, Bethesda, USA) and expressed as a percentage of the gel surface at T = 0.

A subset of gels containing myofibroblasts from the ALDDFT of one foal at T = 24 h and incubated with DMEM, incyclinide, ilomastat, BAPN 3000 µg/mL and DMSO 1% were assessed for cell morphology by phase-contrast microscopy using a Zeiss Axio Vert.A1 inverted phase-contrast microscope (Carl Zeiss AG, Oberkochen, Germany).

### Statistical analysis

Statistical analysis was performed with the statistical software SAS^®^, Version 9.4M7 through SAS Studio 3.81 (SAS Institute Inc., Cary, NC, USA). Assessment of normal distribution was done by the Shapiro-Wilk test and visual assessment of QQ-plots of the model residuals. Variance homogeneity between gels seeded with SDFT and ALDDFT cells was assessed through Pitmann-Morgan-Test and Bonett-Seier-Test for paired samples. Effects of groups over time were calculated by means of a two-way analysis of variance with repeated measurements, taking into account the interaction and a post hoc least square difference test for multiple pairwise comparisons between groups at each time point. The “Mixed” procedure of SAS was used for calculating the linear model. Statistical significance was set at a p-value of ≤ 0.05. *p* ≤ 0.01 was considered very significant, and *p* ≤ 0.001 was considered highly significant. Graphs were created using GraphPad Prism (GraphPad Software, San Diego, CA, USA).

## Results

### Cell culture experiments

**Fig. 3:**
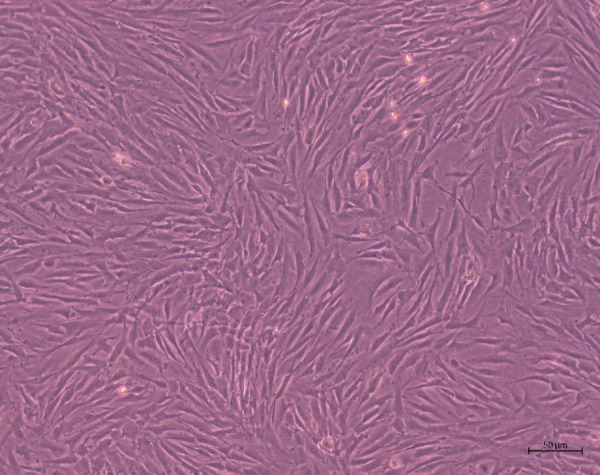
Representative phase contrast micrograph of cultured myofibroblasts from the superficial digital flexor tendon, scale bar = 50 μm.

The microscopic appearance (Fig. 3) of the cultured cells from both the SDFT and the ALDDFT presented a morphology that coincided with that previously described by the authors^13^ and was consistent with human and equine tendon/ligament myofibroblasts in cell cultures^28,29^. They exhibited a fusiform, fibroblast-like morphology, with several membrane protrusions.

### Collagen gel contraction assay

Variance homogeneity between SDFT and ALDDFT was accepted for all substances except BAPN 3000 µg/mL. No significant difference was found between SDFT and ALDDFT for any substance at any time point. Furthermore, gels containing cells from SDFT and ALDDFT were statistically evaluated separately and together as a single uniform sample group. Complete data for ALDDFT and SDFT are shown in Supplementary Tables S1 and S2. Results below refer to the statistical evaluation of SDFT and ALDDFT together, unless stated otherwise.

**Fig. 4:**
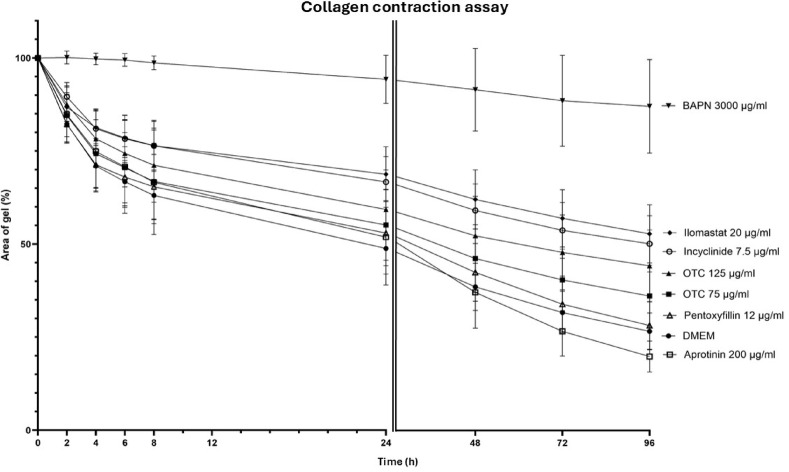
Development over time of the mean (± SD) area of collagen gels (%) seeded with juvenile equine myofibroblasts and incubated with Dulbecco’s modified eagle medium (DMEM), oxytetracycline (OTC) (75 µg/mL; 125 µg/mL), incyclinide (7.5 µg/mL), ilomastat (20 µg/mL), aprotinin (200 µg/mL), pentoxifylline (12 µg/mL) and β-aminopropionitrile fumarate (BAPN) (3000 µg/mL).

BAPN induced the most pronounced inhibition of collagen gel contraction by myofibroblasts (mean 87.04% of the initial gel surface after 96 h) compared to DMEM (mean 26.60% of the initial surface after 96 h), followed by ilomastat (mean 52.81% after 96 h) and incyclinide (mean 50.11% after 96 h), as shown in Fig. 4.

**Fig. 5:**
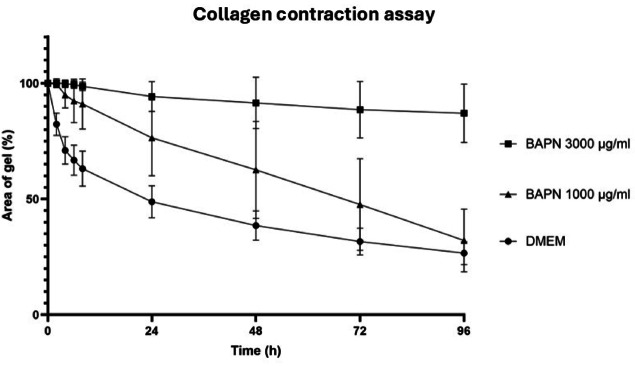
Development over time of the mean (± SD) area of collagen gels (%) seeded with juvenile equine myofibroblasts and incubated with Dulbecco’s modified eagle medium (DMEM), β-aminopropionitrile fumarate (BAPN) (3000 µg/mL) and β-aminopropionitrile fumarate (BAPN) (1000 µg/mL).

In comparison to DMEM, BAPN 3000 µg/mL induced a highly significant inhibition (*p* ≤ 0.001) of collagen gel contraction by cultured myofibroblasts at all time points as presented in Table 2. At a concentration of 1000 µg/mL, BAPN caused a significant inhibition of collagen gel contraction compared to DMEM at T = 2, 4, 6, 8, 24, 48 and 72 but not at T = 96. The inhibition was also significantly weaker than BAPN 3000 µg/mL at all time points except T = 2 and 4, as illustrated in Fig. 5.

**Table:** Mean area (and standard deviation) of collagen gels (%) at Timepoint (T) = 0, 2, 4, 6, 8, 24, 48, 72 and 96 after release, expressed in relation to T0.

	T 0	T 2	T 4	T 6	T 8	T 24	T 48	T 72	T 96
DMEM	100 (0)	82.29^a, b, c^ (4.81)	71.00^a, b, c, d, e, f^ (5.95)	66.79^a, b, c, d, e^ (6.50)	63.08^a, b, c, d, e^ (7.57)	48.83^a, b, c, d, e, f^ (6.91)	38.57^a, b, c, d, e, f^ (6.34)	31.66^a, b, c, d, e, f^ (5.74)	26.60^a, b, c, d, e^ (4.95)
Aprotinin (200 µg/mL)	100 (0)	84.81^d, e^ (7.71)	74.88^g, h^ (10.86)	70.77^f, g, h^ (12.50)	66.62^f, g, h, i^ (14.04)	51.88^g, h, i, j, k^ (12.84)	37.09^g, h, i, j, k, l^ (9.63)	26.62^g, h, i, j, k, l, m^ (6.64)	19.85^f, g, h, i, j, k^ (4.16)
Pentoxifylline (12 µg/mL)	100 (0)	82.22^f, g, h^ (4.84)	71.33^i, j, k, l, m^ (7.04)	68.01^i, j, k, l, m^ (8.14)	65.40 ^j, k, l, m^ (8.70)	52.98^l, m, n, o, p^ (8.74)	42.46^m, n, o, p, q^ (7.76)	33.90^n, o, p, q, r^ (7.56)	28.17^l, m, n, o, p^ (6.40)
Oxytetracycline (75 µg/mL)	100 (0)	84.57^i, j^ (5.71)	74.30^n, o, p^ (9.13)	70.52^n, o, p, q^ (9.46)	66.83^n, o, p, q^ (10.25)	55.14^q, r, s, t^ (9.48)	46.18^r, s, t, u, v^ (9.07)	40.41^g, s, t, u, v^ (8.29)	36.09^f, q, r, s, t^ (8.24)
Oxytetracycline (125 µg/mL)	100 (0)	87.57^k, l^ (4.59)	78.33^a, q, r^ (7.51)	74.39^r, s^ (9.04)	71.18^r, s^ (9.97)	59.29^a, u, v, w^ (10.64)	52.29^a, g, w^ (10.58)	47.83^a, h, n, w^ (10.07)	44.21^a, g, l, u, v^ (9.63)
Incyclinide (7.5 µg/mL)	100 (0)	89.57^a, g, m, n^ (3.85)	81.04^b, i, s, t^ (5.29)	78.32^a, i, n, t, u^ (6.32)	76.42^a, f, j, n, t^ (6.87)	66.69^b, g, l, q, x^ (6.82)	59.09^b, h, m, r, x^ (7.13)	53.74^b, i, o, s, x^ (7.51)	50.11^b, h, m, q, w, x^ (7.58)
Ilomastat (20 µg/mL)	100 (0)	86.81^o, p^ (3.87)	81.31^c, j, n, u, v^ (4.81)	78.54^b, f, j, o, v, w^ (6.07)	76.46^b, g, k, o, u^ (6.54)	68.79^c, h, m, r, u, y^ (7.39)	62.05^c, i, n, s, y^ (7.94)	56.97^c, j, p, t, y^ (7.72)	52.81^c, i, n, r, y, z^ (7.82)
BAPN (1000 µg/mL)	100 (0)	99.66^b, d, f, i, k, m, o, q^ (1.52)	94.95^d, g, k, o, q, s, u, w^ (5.59)	92.37^c, g, k, p, r, t, v, x^ (9.36)	91.03^c, h, l, p, r, v, w^ (10.78)	76.45^d, i, n, s, v, z^ (16.41)	62.62^d, j, o, t, z^ (20.96)	47.63^d, k, z^ (19.76)	32.09^u, w, y, α, β^ (13.52)
BAPN (3000 µg/mL)	100 (0)	100.14^c, e, h, j, l, n, p, r^ (1.74)	99.77^e, h, l, p, r, t, v, x^ (1.58)	99.46^d, h, l, q, s, u, w, x, y^ (1.70)	98.69^d, i, m, q, s, t, u, v, x^ (1.84)	94.29^e, j, o, t, w, x, y, z, α^ (6.46)	91.52^e, k, p, u, w, x, y, z, α^ (11.09)	88.57^e, l, q, u, w, x, y, z, α^ (12.22)	87.04^d, j, o, s, v, x, z, α, γ^ (12.58)
DMSO 1%	100 (0)	89.52^q, r^ (3.08)	84.36^f, m, w, x^ (6.06)	82.66^e, m, y^ (6.91)	78.76^e, w, x^ (9.70)	69.50^f, k, p, α^ (11.07)	62.74^f, l, q, v, α^ (10.61)	57.00^f, m, r, v, α^ (9.63)	54.03^e, k, p, t, β, γ^ (9.76)
DMSO^1^ 0.375%	100	84.65	75.70	72.34	70.51	54.04	48.85	40.55	35.58

Gels were seeded with juvenile equine myofibroblasts from the accessory ligament of the deep digital flexor tendon (ALDDFT) and the superficial digital flexor tendon (SDFT) and incubated with Dulbecco’s modified eagle medium (DMEM), aprotinin (200 µg/mL), pentoxifylline (12 µg/mL), oxytetracycline (OTC) (75 µg/mL; 125 µg/mL), incyclinide (7.5 µg/mL), ilomastat (20 µg/mL), β-aminopropionitrile fumarate (BAPN) (3000 µg/mL; 1000 µg/mL) and DMSO (1%; 0.375%); different superscript letters (a-z, α, β and γ) indicate significant pairwise differences of mean values (p ≤ 0.05) between substances in post hoc least square difference test; ^1^statistical analysis not applicable as N = 1.

In contrast to DMEM, incyclinide induced a significant inhibition (*p* = 0.014) of collagen gel contraction at T = 2 and a highly significant inhibition (*p* ≤ 0.001) of collagen gel contraction at all other time points (Fig. 4; Table 2). The difference failed to reach significance at T = 4, evaluating gels seeded with SDFT only (Supplementary Table S1).

Compared to DMEM, ilomastat induced a highly significant inhibition (*p* ≤ 0.001) of collagen gel contraction by cultured myofibroblasts at all time points, except at T = 2 (Fig. 4; Table 2).

OTC at 75 µg/mL did not cause any significant inhibition of collagen gel contraction compared to DMEM. At a concentration of 125 µg/mL, a significant difference in gel contraction was observed at T = 4 (*p* = 0.0475), T = 24 (*p* = 0.0405), T = 48 (*p* = 0.006), T = 72 (*p* = 0.0005), and T = 96 (*p* ≤ 0.0001) compared with DMEM (Fig. 4; Table 2).

Aprotinin and pentoxifylline did not significantly inhibit collagen gel contraction at the tested concentrations compared with DMEM (Table 2). Significant differences between substances are indicated in Table 2.

At a concentration of 1%, DMSO alone showed significant inhibition of collagen gel contraction compared to DMEM at all time points except at T = 2, as shown in Table 2. There was no significant difference between ilomastat and DMSO 1% at any time point. Incubated with DMSO at 0.375%, collagen gels showed markedly smaller gel surface area than with incyclinide at all time points (Table 2). As only a single biological replicate was tested for DMSO 0.375%, statistical testing could not be performed.

During phase-contrast microscopy, myofibroblasts seeded with BAPN at 3000 µg/mL showed a rounded appearance. In contrast, the morphology of myofibroblasts incubated with DMEM and other substances (incyclinide, ilomastat, DMSO 1%) was fibroblast-like, i.e. spindle-shaped and elongated.

## Discussion and conclusion

Reducing the use of antimicrobials, particularly for non-antimicrobial treatments, has become a priority in veterinary medicine and public health^10,30^. Finding an alternative substance to OTC, lacking antimicrobial properties, for the treatment of flexural limb deformities in foals is of ethical, clinical, and economic importance. The authors began addressing this issue by demonstrating the biocompatibility of potential alternative substances that share properties with OTC, including BAPN, ilomastat, incyclinide, aprotinin, and pentoxifylline^13^. Collagen gels seeded with juvenile equine myofibroblasts have the potential to mimic the basic composition of a tendon matrix and the myofibroblast-mediated tractional structuring of collagen *in vitro*, which is of particular interest for the goal of replacing, reducing, and refining the use of animal experiments^31^. Consequently, to further research alternatives to OTC by evaluating their relaxing potential on the muscle-tendon unit of foals, this study investigated the effect of these substances *in vitro* on the contraction of collagen gels seeded with juvenile equine myofibroblasts.

Among the tested substances, BAPN, at a concentration of 3000 µg/mL, caused the most substantial inhibition of collagen gel contraction at all time points. Moreover, supplementary experiments with BAPN at 1000 µg/mL showed that its effect on collagen gel contraction was concentration-dependent, with lower concentrations exhibiting time-dependent, reversible inhibition of contraction. Caution must be taken when interpreting the statistical results of these supplementary experiments, given the lower number of biological replicates. The evaluation of the cell morphology in a subset of collagen gels revealed a rounded appearance of the myofibroblasts seeded in collagen gels and incubated with BAPN at 3000 µg/mL, compared with a spindle-shaped, elongated fibroblast-like morphology of myofibroblasts incubated with DMEM and other substances. From this, three potential explanations could be deduced. First, it could be inferred that inhibition of collagen gel contraction, through inhibition of collagen cross-linking, may lead to a less elongated cell morphology via mechanical pathways. Moreover, cell morphology also reflects metabolic activity. This aligns with a study examining the effects of BAPN on equine tendon metabolism *in vitro*, which reported similar alterations in cell morphology accompanied by decreased collagen synthesis^21^. Several studies have linked rounded human and equine tendon cell morphology to decreased viability when exposed to substances such as mepivacaine or high-glucose solutions^32,33^. However, as reported by the authors in a previous study, BAPN did not exhibit any cytotoxic or anti-proliferative effects on juvenile myofibroblasts at the tested concentrations^13^. The cells used in MTS assays to assess their viability were incubated with BAPN for 24 h just before reaching 100% confluence. By contrast, the cells used in the current study were seeded in gels that already contained the substance. Consequently, it cannot be ruled out that early exposure to BAPN during myofibroblast growth could have negatively affected their viability, as this specific setting was not tested in this study and a preliminary study^13^. If the viability of the myofibroblasts was impaired in this setting, the process appears, however, to be reversible at lower concentrations, as demonstrated by the effect of BAPN 1000 µg/mL on the collagen gel contraction. Finally, rounded nuclei morphology was associated with increased metabolic activity in an explant study and was observed in the early tendon-repair phase following degenerative lesions in another study^34,35^. Although this latter explanation is less likely, it cannot be excluded that increased metabolic activity caused the rounded morphology of the studied cells. Consequently, while BAPN shows promising results in this collagen gel contraction assay, further research should investigate the dose-dependent effect described here to minimise potential adverse effects on cell morphology.

BAPN is the toxic constituent of the sweet pea plant (*Lathyrus odoratus*) and a lysyl oxidase inhibitor, causing inhibition of collagen cross-linking^21,26,36^. In equine veterinary medicine, BAPN has been used intralesionally to treat tendinopathies^26,37^. The proposed mechanism of action in tendinopathies is a delay in collagen cross-linking in scar tissue, which encourages the parallel alignment of collagen fibres following loading of the tendon^21^. Some inflammatory reactions at the injection site have been described, and evidence of tendon healing is controversial; some studies report a decrease in collagen production^21^ while others report a reduced risk of scar formation through better alignment of collagen fibrils^26,38^. Systemic administration has not been described in equine patients and entails risks, as BAPN has been shown to cause skeletal pathologies (osteolathyrism) in immature rats after systemic administration^36^. Local intra-or peri-tendinous applications or administration through intravenous regional perfusion to limit the systemic dose while maximising the local effect of BAPN could be investigated as potential routes of administration in clinical settings for juvenile equine patients.

The MMP-inhibitors ilomastat and incyclinide also showed significant inhibition of collagen gel contraction, making them interesting candidates as alternatives to OTC. These substances, being lipophilic, were dissolved in diluted DMSO at concentrations of 1% and 0.375%, respectively, for ilomastat and incyclinide. The inhibition of collagen contraction caused by DMSO alone at a concentration of 1% was similar to that of ilomastat in this study, raising the question of which part of the inhibition of collagen gel contraction was driven by ilomastat and which by DMSO. DMSO has been used for its anti-inflammatory and free-oxygen-radical scavenging properties to treat young foals, such as those with maladjustment syndrome, without a documented relaxing effect on the muscle-tendon unit^39^. Detrimental effects of DMSO on cell viability have been described in various cell populations *in vitro* in a dose-dependent manner^40^. However, the effect of 1% DMSO on collagen gel contraction by myofibroblasts through an alteration of myofibroblast viability is unlikely, as the authors did not find any cytotoxic or anti-proliferative effects of 1% DMSO on juvenile equine myofibroblasts in a previous study^13^. Although tested in only 1 biological replicate in this study, which makes this result anecdotal, 0.375% DMSO did not appear to inhibit collagen gel contraction to the same extent as incyclinide. This suggests that the effect of incyclinide was not substantially influenced by its dilution in DMSO. Further experiments with DMSO at varying concentrations or using alternative solvents are needed to fully assess the potential of ilomastat.

Incyclinide (COL-3, CMT-3) is one of the eight chemically modified tetracyclines (CMTs) initially developed and is among the most studied and promising, for example, in the field of human oncology^2,41^. Apart from their absence of antimicrobial properties, CMTs exhibit several advantages over conventional tetracyclines in humans, including preserved or enhanced MMP-inhibitor properties, a lack of described gastrointestinal toxicity, and higher achievable plasma concentrations^2^. Although incyclinide is a relatively broad-spectrum MMP-inhibitor, it has shown specificity for the gelatinases MMP-2 and MMP-9^1,2,41^. Arnoczky et al. (2004) reported that OTC induced a decrease of MMP-1 mRNA expression by equine myofibroblasts^6^. Assuming a specific role of MMP-1 in the relaxing effect of OTC on the muscle-tendon unit, further CMTs could be examined for their biocompatibility with equine myofibroblasts and their effects in collagen gel contraction assays and compared to incyclinide. CMTs have been studied, among others, in the field of oncology for oral administration^2,41^. The pharmacokinetics of CMTs are characterized by their hydrophobic nature, which poses challenges for their administration, particularly as an intravenous route of application would potentially be desirable for an alternative to OTC^2,41^. In human trials for cancer treatment, phototoxicity has been the primary adverse reaction reported^41^, which could potentially be a concern in applications in young foals. Consequently, following *in vitro* experiments, incyclinide represents an interesting potential alternative to OTC; however, further research is needed to describe its pharmacokinetics and detect potential side effects in juvenile equine patients.

In this study, OTC did not significantly inhibit collagen gel contraction by equine myofibroblasts at a concentration of 75 µg/mL compared to DMEM, although a trend was observed. This contrasts with the results of another study, which reported that OTC inhibited collagen gel contraction, finding a significant inhibition at a concentration of 75 µg/mL but not consistently at concentrations of 12.5–25 µg/mL^6^. In our study, significance was achieved at a concentration of 125 µg/mL at T = 4, 24, 48, 72, and 96 h. Although our protocol was similar to that described by Arnoczky et al. (2004) in some respects (collagen and cell concentrations), some other aspects differed^6^. Mainly, the collagen gels in the current study were placed in 24-well plates instead of 60-mm culture dishes, and rat tail collagen was used instead of bovine collagen. These technical differences or unobserved factors could have influenced the results. Given the clinical efficacy of OTC, the weak effect observed at 75 µg/mL in these *in vitro* experiments may be a limitation of the model. The significant inhibition observed at higher dosages, however, tends to underscore the difficulty of adapting clinical dosages to in vitro experiments rather than a critical translatability weakness. The significant inhibition of collagen gel contraction obtained in this study after incubation with BAPN and incyclinide, as described above, generally suggests a more substantial effect of these substances on the muscle-tendon unit compared to OTC.

Aprotinin and pentoxifylline did not significantly inhibit the contraction of collagen gels at any time points compared to DMEM. Although these substances possess MMP-inhibitor properties, they did not demonstrate the same effect as incyclinide at the tested concentration. It is possible that the chosen concentrations were not optimal, and higher concentrations might have yielded different results.

The *in vitro* nature of this research limits the ability to draw direct clinical conclusions. As the collagen gels in this study mimic the basic composition of a juvenile equine tendon, and myofibroblasts exert tractional structuring, thus contracting the gels, the inhibition of contraction serves as a model for the inhibition of tractional structuring and collagen matrix organisation in juvenile tendons and ligaments. This is potentially caused by MMP inhibition^6^ or the inhibition of collagen cross-linking by BAPN^7^. The hypothesis underlying these mechanisms is that the resulting inhibition of collagen alignment increases tendon susceptibility to time-dependent strain or creep^6^. However, fascicular organization, cross-link maturation, neuromuscular input, mechanical loading and physiological strain patterns are not considered in this model, making direct extrapolation to a tendon in a living animal impossible.

In conclusion, BAPN and incyclinide showed a significant inhibitory capacity on collagen gel contraction by juvenile equine myofibroblasts, which indicates a potential relaxing effect on the muscle-tendon unit of foals. Consequently, they deserve to be studied further in search of potential substitutes for OTC. Moreover, further investigations into their side effects, pharmacodynamics, pharmacokinetics, and methods of administration in juvenile equine patients are needed to translate these promising *in vitro* results into clinical applications.

## Supplementary Information

Supplementary Information.

## Data Availability

The datasets generated and analysed during the current study are available from the corresponding author on reasonable request.
