# Supplementary material for: An in vitro collagen gel contraction assay to assess the relaxing effect of potential pharmacological alternatives to oxytetracycline on foals’ tendons
Source: Sci Rep. 2026 Apr 25;16:13412. doi: 10.1038/s41598-026-49449-4 (PMC13110344; doi:10.1038/s41598-026-49449-4)
Supplement: Supplementary file 1 — Supplementary Information. [file 41598_2026_49449_MOESM1_ESM.docx]

|  | **T 0** | **T 2** | **T 4** | **T 6** | **T 8** | **T 24** | **T 48** | **T 72** | **T 96** |
| --- | --- | --- | --- | --- | --- | --- | --- | --- | --- |
| DMEM | 100  (0) | 82.50^a, b, c^  (3.12) | 72.40^a, b, c^  (3.23) | 67.86^a, b, c, d, e^  (5.90) | 64.65^a, b, c, d^  (6.09) | 50.10^a, b, c, d, e^  (5.54) | 39.54^a, b, c, d^  (7.08) | 31.81^a, b, c, d, e^  (7.10) | 25.51^a, b, c, d, e^  (5.87) |
| Aprotinin  (200 µg/mL) | 100  (0) | 85.03^d, e^  (6.51) | 75.28^d, e^  (10.51) | 72.42 ^f, g^  (11.27) | 67.82 ^e, f^  (12.91) | 52.32^f, g, h, i^  (12.19) | 39.12^e, f, g, h^  (8.83) | 28.54^f, g, h, i, j, k^  (6.03) | 20.54^f, g, h, i, j, k^  (4.56) |
| Pentoxifylline  (12 µg/mL) | 100  (0) | 81.97^f, g, h^  (4.79) | 72.01^f, g, h, i^  (5.18) | 69.25^h, i, j^  (6.85) | 66.02 ^g, h, i^  (7.72) | 54.19^j, k, l^  (8.97) | 43.54^i, j, k^  (9.35) | 34.97^l, m, n, o^  (9.04) | 28.38^l, m,^ ^n, o, p^  (7.83) |
| Oxytetracycline  (75 µg/mL) | 100  (0) | 83.89^i, j^  (6.34) | 74.58^j, k^  (9.80) | 71.26^k, l^  (9.38) | 67.65^j, k^  (10.48) | 56.48 ^m, n^  (10.52) | 47.86^l, m^  (11.06) | 41.97^f, p, q^  (10.27) | 36.91^f, q, r^  (10.59) |
| Oxytetracycline  (125 µg/mL) | 100  (0) | 86.46^k, l^  (3.59) | 77.85^l, m^  (5.53) | 74.50^m^  (8.74) | 71.33^l^  (10.31) | 61.31 ^o^  (12.16) | 54.07^n, o^  (12.08) | 49.88^a, g, r^  (12.29) | 45.66^a, g, l, s, t^  (11.35) |
| Incyclinide  (7.5 µg/mL) | 100  (0) | 88.98^a, g, m^  (3.62) | 80.33^f, n^  (5.88) | 77.62^a, n^  (6.43) | 75.70^a, m^  (8.03) | 66.70^a, f, j, p^  (8.21) | 58.50^a, e, i, p^  (9.15) | 53.01^b, h, l, s, t^  (10.30) | 48.21^b, h, m, u, v^  (10.00) |
| Ilomastat  (20 µg/mL) | 100  (0) | 86.12^n, o^  (3.43) | 81.02^a, g, o^  (5.43) | 79.24^b, h, o^  (6.35) | 77.02^b, g, n^  (6.85) | 70.78^b, g, k, m, q^  (7.51) | 64.14^b, f, j, l, n, q^  (8.19) | 59.22^c, i, m, p, u, v^  (8.48) | 54.66^c, i, n, q, w, x^  (8.70) |
| BAPN  (1000 µg/mL) | 100  (0) | 98.51^b, d, f, i, k, n, p^  (1.20) | 92.17^b, d, h, j, l, p^  (6.07) | 89.20^c, f, i, k, p^  (10.64) | 87.33^c, e, h, j, o^  (12.93) | 74.43^c, h, r^  (16.80) | 59.79 ^r^  (18.15) | 41.82^s, u, w^  (15.15) | 27.01^s, u, w, x, y^  (13.35) |
| BAPN  (3000 µg/mL) | 100  (0) | 99.53^c, e, h, j, l, m, o, q^  (1.14) | 99.26^c, e, i, k, m, n, o, p, q^  (1.27) | 99.04^d, g, j, l, m, n, o, p, q^  (1.58) | 98.38^d, f, i, k, l, m, n, o, p^  (2.30) | 92.67^d, i, l, n, o, p, q, r, s^  (8.12) | 87.30^c, g, k, m, o, p, q, r, s^  (13.16) | 83.96^d, j, n, q, r, t, v, w, x^  (14.20) | 81.37^d, j, o, r, t, v, x, x, z^  (13.79) |
| DMSO  1% | 100  (0) | 90.15^p, q^  (1.41) | 85.92^q^  (1.68) | 84.57 ^e, q^  (1.19) | 81.01^p^  (1.16) | 72.21^e, s^  (3.20) | 64.43^d, h, s^  (3.99) | 58.78^e, k, o, x^  (4.82) | 55.03^e, k, p, y, z^  (7.31) |

**Supplementary Information**

**Supplementary Table S1:** Mean area (and standard deviation) of collagen gels (%) at Timepoint (T) = 0, 2, 4, 6, 8, 24, 48, 72 and 96 after release, expressed in relation to T0. Gels were seeded with juvenile equine myofibroblasts from the superficial digital flexor tendon (SDFT) and incubated with Dulbecco’s modified eagle medium (DMEM), aprotinin (200 µg/mL), pentoxifylline (12 µg/mL), oxytetracycline (OTC) (75 µg/mL; 125 µg/mL), incyclinide (7.5 µg/mL), ilomastat (20 µg/mL), β-aminopropionitrile fumarate (BAPN) (3000 µg/mL; 1000 µg/mL) and DMSO (1%); different superscript letters (a-z) indicate significant pairwise differences of mean values (p ≤ 0.05) between substances in post hoc least square difference test

|  | **T 0** | **T 2** | **T 4** | **T 6** | **T 8** | **T 24** | **T 48** | **T 72** | **T 96** |
| --- | --- | --- | --- | --- | --- | --- | --- | --- | --- |
| DMEM | 100  (0) | 82.07^a, b, c^  (6.41) | 69.60^a, b, c, d, e^  (7.93) | 65.72^a, b, c, d^  (7.45) | 61.51^a, b, c, d^  (9.12) | 47.56^a, b, c, d^  (8.39) | 37.59^a, b, c, d, e^  (5.99) | 31.52^a, b, c, d, e, f^  (4.70) | 27.70^a, b, c, d, e^  (4.07) |
| Aprotinin  (200 µg/mL) | 100  (0) | 84.59^d, e^  (9.40) | 74.47^f, g^  (12.18) | 69.12^e, f^  (14.49) | 65.43^e, f, g, h^  (16.24) | 51.44^e, f, g, h^  (14.62) | 35.06^f, g, h, i, j, k^  (10.78) | 24.71^g, h, i, j, k, l, m^  (7.20) | 19.17^f, g, h, i, j, k, l^  (4.02) |
| Pentoxifylline  (12 µg/mL) | 100  (0) | 82.47^f, g, h^  (5.33) | 70.66^h, i, j, k^  (9.00) | 66.78^g, h, i, j^  (9.75) | 64.78 ^i, j, k, l^  (10.30) | 51.77^i, j, k, l^  (9.18) | 41.38^l, m, n, o^  (6.49) | 32.84^n, o, p, q, r^  (6.42) | 27.95^m, n, o, p, q^  (5.35) |
| Oxytetracycline  (75 µg/mL) | 100  (0) | 85.25^i, j^  (5.51) | 74.02^l, m, n^  (9.34) | 69.79 ^k, l^  (10.36) | 66.00^m, n, o^  (10.94) | 53.80^m, n, o, p^  (9.09) | 44.51^p, q, r^  (7.20) | 38.86^g, s, t, u^  (6.33) | 35.23^f, r, s, t, u^  (5.97) |
| Oxytetracycline  (125 µg/mL) | 100  (0) | 88.49^k, l^  (5.44) | 78.72^a,^ ^o, p^  (9.38) | 74.31^m, n^  (10.11) | 71.06^p, q^  (10.66) | 57.61^q, r^  (10.03) | 50.80^f, s^  (10.05) | 46.13^a, h, v^  (8.61) | 43.00^a, g, m, v^  (8.86) |
| Incyclinide  (7.5 µg/mL) | 100  (0) | 90.16^a, g, m^  (4.31) | 81.75^b, h, q^  (5.07) | 79.02^a, g, o^  (6.75) | 77.13^a, e, i, m, r^  (6.17) | 66.70^a, e, i, m, s^  (5.91) | 59.68^a, g, l, p, t^  (5.21) | 54.47^b, i, n, s, w^  (4.07) | 52.01^b, h, n, r, w, x^  (4.22) |
| Ilomastat  (20 µg/mL) | 100  (0) | 87.51^n, o^  (4.48) | 81.60^c, i, l, r, s^  (4.62) | 77.84^b, h, p, q^  (6.28) | 75.90^b, f, j, s^  (6.82) | 66.81^d,^ ^f, j, n, t^  (7.36) | 59.97^b, h, m, q, u^  (7.83) | 54.71^c, j, o, t, x^  (6.85) | 50.97^c, i, o, s, y, z^  (7.12) |
| BAPN  (1000 µg/mL) | 100  (0) | 100.81^b, d, f, i, k, n, p^  (0.60) | 97.74^d, f, j, m, o, r, t^  (4.23) | 95.54^c, e, i, k, m, p, r^  (8.70) | 94.74^c, g, k, n, p, t^  (9.07) | 78.47^b, g, k, o, q, u^  (19.45) | 65.44^c, i, n, v^  (27.29) | 53.44^d, k, y, r^  (25.41) | 37.17^l, w, y, α^  (14.19) |
| BAPN  (3000 µg/mL) | 100  (0) | 100.75^c, e, h, j, l, m, o, q^  (2.11) | 100.27^e, g, k, n, p, q, s, u^  (1.80) | 99.89^d, f, j, l, n, o, q, s^  (1.86) | 99.00^d, h, l, o, q, r, s, u^  (1.37) | 95.91^c, h, l, p, r, s, t, u, v^  (4.42) | 95.75^d, j, o, r, s, t, u, v, w^  (7.37) | 93.17^e, l, p, u, v, w, x, y, z^  (8.73) | 92.71^d, j, p, t, v, x, z, α, β^  (9.00) |
| DMSO  1% | 100  (0) | 88.89^p, q^  (4.52) | 82.80^t, u^  (9.03) | 80.75^r, s^  (10.35) | 76.52 ^t, u^  (14.80) | 66.80^v^  (16.56) | 61.04^e, k, w^  (16.03) | 55.21^f, m, q, z^  (14.11) | 53.04^e, k, q, u, β^  (13.47) |
| DMSO^1^  0.375% | 100 | 84.65 | 75.70 | 72.34 | 70.51 | 54.04 | 48.85 | 40.55 | 35.58 |

**Supplementary Table S2:** Mean area (and standard deviation) of collagen gels (%) at Timepoint (T) = 0, 2, 4, 6, 8, 24, 48, 72 and 96 after release, expressed in relation to T0. Gels were seeded with juvenile equine myofibroblasts from the accessory ligament of the deep digital flexor tendon (ALDDFT) and incubated with Dulbecco’s modified eagle medium (DMEM), aprotinin (200 µg/mL), pentoxifylline (12 µg/mL), oxytetracycline (OTC) (75 µg/mL; 125 µg/mL), incyclinide (7.5 µg/mL), ilomastat (20 µg/mL), β-aminopropionitrile fumarate (BAPN) (3000 µg/mL; 1000 µg/mL) and DMSO (1%; 0.375%); different superscript letters (a-z, α and β) indicate significant pairwise differences of mean values (p ≤ 0.05) between substances in post hoc least square difference test; ^1^statistical analysis not applicable as N = 1
